# Constant light exposure and/or pinealectomy increases susceptibility to trichloroethylene-induced hepatotoxicity and liver cancer in male mice

**DOI:** 10.1007/s11356-022-19976-4

**Published:** 2022-04-14

**Authors:** Mohamed E. Abdraboh, Mohamed A. El-Missiry, Azza I. Othman, Ahmed Nageeb Taha, Dalia S. Abd Elhamed, Maggie E. Amer

**Affiliations:** 1 Zoology Department, Faculty of Science, Mansoura University, Mansoura, Egypt; 2 Neurosurgery Department, Faculty of Medicine, Mansoura University, Mansoura, Egypt; 3 Faculty of Science, New Mansoura University, Mansoura, Egypt

**Keywords:** Trichloroethylene, Pollution, Melatonin, Liver cancer, Constant light, Circadian rhythm, Oxidative stress

## Abstract

Exposure to light at night, pineal gland impairment, and the environmental pollutant trichloroethylene (TCE) have serious implications for health and contribute to illness, including liver cancer. The adverse effect of the association of continuous exposure to light with decreased melatonin levels and TCE-induced toxicity is not disclosed in target organs. This work explored the role of light and pineal impairment in increasing susceptibility to liver toxicity and cancer upon exposure to TCE. Male albino mice were divided into groups as follows: control group (12-h light/12-h dark cycle), constant light (24-h light), pinealectomized (Pnx) mice, sham surgically treated group, TCE-treated groups subjected to two doses (500 and 1000 mg/kg) at two different light regimens, and combination of Pnx and TCE-treated mice kept at a 12-h light/12-h dark cycle. Melatonin levels were significantly decreased in both Pnx mice and TCE-treated animals at both light regimens. Aspartate transaminase, alanine aminotransferase, activities, and serum bilirubin levels were significantly elevated, whereas albumin levels were markedly decreased in Pnx mice, TCE-treated mice, and the combination group. Histopathological investigations reflected changes in liver function parameters indicating liver injury and induction of cancer. These effects were accompanied by significant increase of the liver cancer biomarker alpha-fetoprotein and the expression of the metastatic markers CD44, TGFβ-1, and VEGF, along with increased oxidative stress indicators and inflammatory cytokines (IL-6, IL-1β, and TNF-α) in both Pnx and TCE-treated mice and the combination group at both light regimens. Taken together, our findings indicated that low melatonin levels, exposure to constant light, and the combination of both factors increases susceptibility to the toxic and carcinogenic effects of TCE on the liver.

## Introduction

Light is a crucial physiological influencer of circadian organization of the organs of the human body and daily activity. Shift work and changes in sleep–wake behavior that increase exposure to light at night are prevalent in most modern societies. People in both categories display disruption in melatonin production, and decreased levels of melatonin release into the blood at night could increase the risk of several disorders and diseases, including cancer (Walker et al. 2020). All mammals possess a circadian timing system that generates 24-h rhythms in many physiological processes (van Zuylen et al. 2021). The rodent and human circadian timing system share many common features.

Melatonin (N-acetyl-5-methoxytryptamine) is a neurohormone synthesized and released primarily by the pineal gland. Melatonin production is enhanced at night and reduced by light at daytime (Zisapel 2018). Disturbance of circadian rhythm can be a result of exposure to artificial light during night time and decreased melatonin synthesis and release due to pineal dysfunction (Burgess and Emens 2020). Night shift workers are highly susceptible to cancer (Touitou et al. 2017). Suppression of melatonin by light during night shift work and due to pineal impairment have remarkable influence on cancer development (Hunter and Figueiro 2017). This is attributed to decreased melatonin levels and disturbed circadian rhythm due to exposure to artificial light at night (Hunter and Figueiro 2017). A number of studies have shown that decreased melatonin levels are associated with the development of several cancers (Carter et al. 2014; Manouchehri et al. 2021; Schernhammer et al. 2003). On the other hand, highly credible evidence reports the anticancer effects of melatonin (Reiter et al. 2017). Several studies have documented the preventive and therapeutic effects of melatonin on the initiation and development of several cancers, which are attributed to its potent antioxidant and free radical scavenging activity (Amin et al. 2019; Reiter et al. 2017; Talib 2018). Additionally, outdoor blue light that is prevalent in recent years has been shown to suppress melatonin and increase cancer risk (Garcia-Saenz et al. 2020). An association between average daily television viewing time and the incidence of ovarian cancer has also been reported (Ukawa et al. 2018). Additionally, increased cancer risk is associated with short sleep duration in Asian populations (Chen et al. 2018).

Trichloroethylene (TCE) is an environmental and occupational contaminant that is produced and applied on a large scale and often disposed inappropriately (Horzmann et al. 2020). The primary sources releasing TCE into the environment are metal cleaning and degreasing operations (Wu and Schaum 2000). It is a well-known carcinogen and is associated with several toxic effects, including immunotoxicity, reproductive toxicity, neurotoxicity, cardiotoxicity (Huang et al. 2021), and teratogenic effects (Huang et al. 2015), and is a potential risk factor in the development of neurodegenerative disorders (De Miranda and Greenamyre 2020). Despite the associations of TCE exposure with many cancers and diseases, the molecular mechanisms of TCE-induced negative health effects are unclear. A recent study reported on the association between TCE exposure and increased DNA methylation of genes encoding markers of autoimmune disease and cancer (Phillips et al. 2019). Pathomechanisms of toxicity include dysfunction of mitochondria and disrupted membrane potential (Elkin et al. 2019), oxidative stress (Elkin et al. 2018), and excessive production of proinflammatory cytokines (Hassan et al. 2016). The effect of low melatonin level and/or constant light exposure on susceptibility of TCE-induced liver cancer development is unclear.

To the best of our knowledge, there have been no studies on the association of continuous exposure to light with decreased melatonin levels and TCE-induced toxicity. This work aimed to evaluate the role of light at night and pineal impairment in increasing the hepatotoxicity and incidence of liver cancer development upon exposure to the environment pollutant TCE.

## Materials and methods

### Chemicals

Melatonin and TCE were obtained from Sigma Chemical Company, USA. All other chemicals were of highest analytical grade and obtained from Al-Gomhoria Chemical Company, Egypt.

### Animals

Adult male albino mice weighing 25–30 g were obtained from the Egyptian Vaccine Company (VACSERA, Giza, Egypt). During the experimental period, mice were given a standard rodent diet, water ad libitum, and maintained at room temperature for a week before the experiment. The experimental protocol for the treatment of mice was performed according to guidelines and approved by the Institutional Animal Ethics of Mansoura University committee (Sc-Z-M-2021–42).

### Animal groups and experimental design

Animals were divided into ten groups, 5 mice each, as follows:

- Group 1 (Control 12-h L): In which mice did not receive any treatment except the vehicle (corn oil) and exposed to 12-h light (L)/12-h dark (D) cycle. Light intensity of 175–200 lx was used. During accommodation and experimentation, light was emitted by white ceiling fluorescent tubes along the middle of the room. The cages were positioned in a manner to ensure medium light intensity average of 175 lx. Light intensity was verified in the animal house with a light meter (Light meter UNI-T UT383S, China). The experimental period was 30 days.
- Group 2 (Control 24-h L): Mice maintained at 24-h L in similar conditions as the Group 1 control group for 30 days at light intensity 175–200 lx.
- Group 3 (Sham): Mice subjected to surgery without removal of the pineal gland. These mice were kept at 12-h L/12-h D cycle, and the light intensity was 175–200 lx during the experimental period.
- Group 4 (Pnx 12-h L): In which the pineal glands were removed surgically as previously described (Maganhin et al. 2009). These mice were kept at 12-h L/12-h D cycle, and the light intensity was 175–200 lx.
- Group 5 (TCE 500 mg, 12-h L): Mice exposed to 12-h L/12-h D cycle and given TCE 500 mg/kg daily by stomach tube for 6 days, then left without treatment till the end of experimental period. TCE was prepared in corn oil.
- Group 6 (TCE 500 mg, 24-h L): Mice exposed to 24-h L and treated with TCE 500 mg/kg daily for 6 days, which was continued till the end of the experimental period of 30 days in constant light.
- Group 7 (Pnx + TCE 500 mg, 12-h L): Pinealectomized animals exposed to 12-h L/12-h D cycle and treated with TCE 500 mg/kg daily for 6 days in a 12-h L/12-h D cycle.
- Group 8 (TCE 1000 mg, 12 h L): Animals exposed to 12-h L/12-h D cycle and treated with TCE 1000 mg/kg daily for 6 days and kept at a 12-h L/12-h D light cycle for 30 days.
- Group 9 (TCE 1000 mg, 24-h L): Mice exposed to 24-h light cycle and treated with TCE 1000 mg/kg daily for 6 days in constant light.
- Group 10 (Pnx + TCE 1000 mg, 12 h L): Pinealectomized animals exposed to a 12-h L/12-h D cycle and treated with TCE 10,000 mg/kg daily for 6 days.

### Surgical procedure for pinealectomy

Pinealectomy was performed as previously described (Maganhin et al. 2009). Briefly, overnight fasted mice were anesthetized with 15-mg/kg xylazine and 30-mg/kg ketamine, then subjected to a longitudinal opening in the scalp to expose the lambda suture. The skull around the lambda suture was carefully removed, followed by the pineal gland removal using fine forceps. The cranium bone was placed back to its original position, and the scalp was sutured. The procedure was completed within 30 min. After surgery, the animals received a single prophylactic antibiotic (amoxicillin) and analgesic (ketoprofen) via the intramuscular route. All operated mice were allowed to recover for 2 weeks before starting the experiment.

### Sample collection

The experiment lasted for 30 days, after which the animals were euthanized with ketamine/xylazine (0.1 ml/100 g, i.p.) for blood and liver collection.

Blood samples were collected directly from hearts into clean tubes. Sera were prepared by centrifugation at 1500 × *g* and then used for biochemical assays. The mice were dissected, and livers were harvested. Portions of the liver were homogenized in cold phosphate buffer, centrifuged at 3000 × *g* to obtain the supernatant, and kept at − 4 °C for biochemical analysis. Other liver portions were fixed for 48 h in 10% buffered neutral formalin (pH 7.4) for histological and immunohistochemical investigation.

### Determination of biochemical parameters in serum

A melatonin ELISA kit obtained from Fine Test, EM1218 Wuhan Biotech, China, was used to determine free melatonin serum levels according to the manufacturer’s instructions. Melatonin levels were expressed as pg/ml. Alfa fetoprotein (AFP) serum levels were also determined through ELISA in accordance with the manufacturer’s instructions using kits obtained from Bio-Techne (MAFP00) Minneapolis, USA. The level of vascular endothelial growth factor (VEGF) in serum was estimated using an ELISA kit (EK0541) obtained from BosterBio, USA.

Cytokines levels were estimated by ELISA kits obtained from My Biosource (San Diego, USA) according to the instruction manual for interleukin (IL)-10 (MBS824703), IL1-β (MBS175967), and IL-10 (MBS704754). Levels of tumor necrosis factor alpha (TNF-a) were evaluated by an ELISA kit (ELM-TNFa) obtained from RayBiotech, GA, USA. Liver function parameters, namely, aspartate transaminase (AST), alanine aminotransferase (ALT), albumin, and bilirubin, were determined calorimetrically in serum according to the instruction manual of the kits obtained from Biodiagnostics, Giza, Egypt. The levels of glutathione (GSH) and activities of glutathione peroxidase (GPx) and glutathione reductase (GR) in the liver were determined in accordance with the instructions in the manual of kits obtained from Biodiagnostics, Egypt. The levels of malondialdehyde (MDA), hydrogen peroxide (H_2_O_2_), and nitric oxide (NO) were determined calorimetrically in liver following the instruction of the kit purchased from Biodiagnostics, Giza, Egypt.

### Histopathology

A standard procedure was adopted to prepare paraffin wax blocks containing liver samples; then, 5-μm sections were prepared. The sections were processed following the standard procedure for hematoxylin and eosin staining for histopathological observation. Stained sections were then observed using an Olympus light microscope and photographed using an Amscope MU1000 camera. The extent of hepatic tissue injury was then evaluated via semiquantitative scoring in five randomly selected fields for each section. The scoring system relied on the tissue involvement percentage, as described (Khafaga and El-Sayed 2018), considering hepatic cords arrangement, sinusoidal dilation and hepatocytic necrosis. Liver injury parameters were scored as none (0): no involvement of evaluated field; mild (1): involvement of 0–25% of evaluated field; moderate (2): involvement of 25–50% of evaluated field; and severe (3): involvement of 50–100% of evaluated field (Khafaga et al. 2019).

### Immunohistochemistry

Liver Sects. (5 μm) were processed for immunohistochemical staining according to streptavidin-peroxidase method. After deparaffinization and hydration, liver sections were washed with phosphate-buffered saline (PBS) 3 times and 3% H2O2 was used to quench endogenous peroxidase activity. After rinsing with PBS, sections were blocked with 5% bovine serum albumin (Cat. #A9647; Sigma Aldrich, USA) diluted in 0.1 M PBS (pH 7.2) for 1 h (Noreldin et al. 2021). The sections were then processed for immunohistochemical staining and incubated overnight at 4 °C with primary antibodies CD44 (156-3C11), monoclonal mouse, Invitrogen, USA (1/400), Catalog # MA5-13,890. For TGF-β, Polyclonal rabbit, Invitrogen, USA (1/100), Catalog #PA1-9574 was used. Bound primary antibodies were localized by applying secondary goat anti-rabbit IgG H&L (HRP), abcam, USA (1:100), Catalog # (ab205718), and development was conducted using diaminobenzidine (DAB) stain according to the manufacturer’s instructions (Vector Lab, Tucson, AZ). An Olympus microscope C31 equipped with an Amscope mu 1000 camera was used to examine the sections under bright field. The labeling index was assessed as described previously (Amer et al. 2021) using Image J software (Anggorowati et al. 2017).

### Statistical analysis

Data were analyzed using the statistical software program Prism (GraphPad, Prism, 6.01). The mean ± standard deviation of each variable was estimated. Two-way ANOVA test was used to compare effect of combined independent factors on single continuous parametric outcome with post hoc Tukey test for pairwise comparison. Differences were considered statistically significant at *P* < 0.05.

## Results

### Comparison of melatonin levels across study groups

**Fig. 1:**
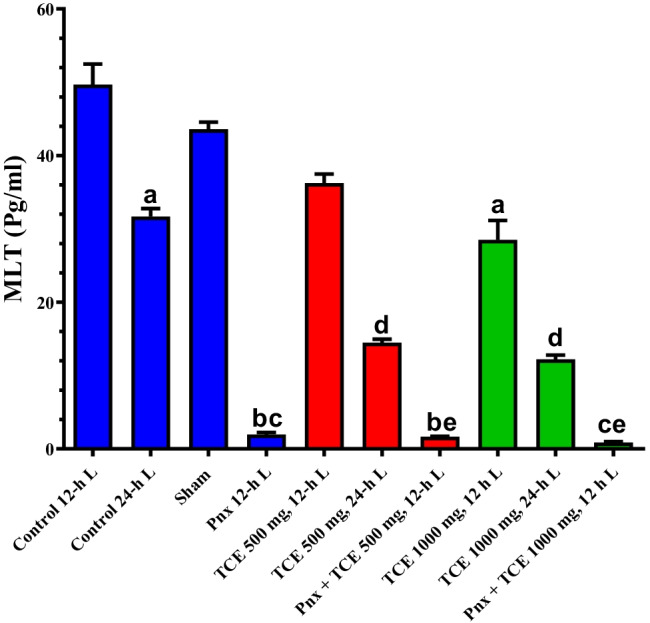
Serum melatonin levels of mice in the control and different treatment groups. Values are expressed as mean ± standard deviation of the mean. Same letters represent non-significant difference between groups. Differences were considered statistically significant at *P* < 0.05

The serum melatonin levels of all animal groups were evaluated and are presented in Fig. 1. The mice exposed to 24-h L showed a significant (*P* < 0.05) decrease in the serum melatonin compared with the control group exposed to the 12-h L/12-h D cycle. The Pnx mice exposed to 12-h L/12-h D cycle exhibited a significant (*P* < 0.05) decrease in melatonin levels in blood. The treatment with either 500 or 1000 mg/kg TCE caused a significant (*P* < 0.05) decrease in melatonin levels in animals exposed to 12-h L/12-h D and 24-h L compared with the control animals. The Pnx mice that received both doses of TCE also exhibited a significant (*P* < 0.05) decrease in blood melatonin compared with the control animals. The sham surgery group showed an insignificant change from the control value.

### Serum levels of liver function parameters, AST, ALT, albumin, and bilirubin

**Fig. 2:**
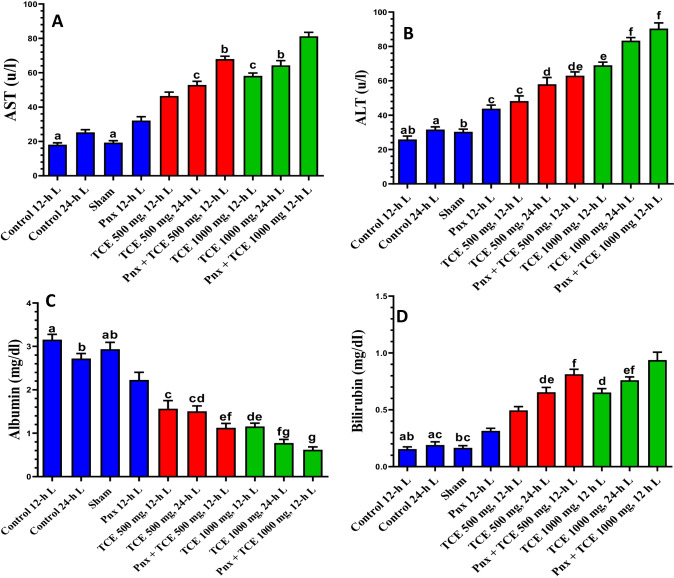
Serum levels of aspartate transaminase (AST) **A**, alanine aminotransferase (ALT) **B**, albumin **C**, and bilirubin **D** of mice in the control and different treatment groups. Values are expressed as mean ± standard deviation of the mean. Same letters represent non-significant difference between groups. Differences were considered statistically significant at *P* < 0.05

We determined expression changes in liver function parameters, including the activity of AST and ALT and levels of albumin and bilirubin in serum, across the study groups (Fig. 2). The data showed an insignificant change in these parameters in serum of mice kept at constant light and sham-operated mice compared with the control (12-h L/12-h D cycle) mice. On the other hand, Pnx mice and TCE-treated mice kept in both light regimens showed a significant (*P* < 0.05) elevation in the activity of ALT and AST and serum bilirubin. Additionally, the albumin concentration in the serum of these rats showed a significant (*P* < 0.05) decrease compared with control animals kept in 12-h L/12-h D cycle. Pnx mice treated with both doses of TCE showed higher values compared with rats treated with TCE at both light regimens.

### Liver histopathology findings

**Fig. 3:**
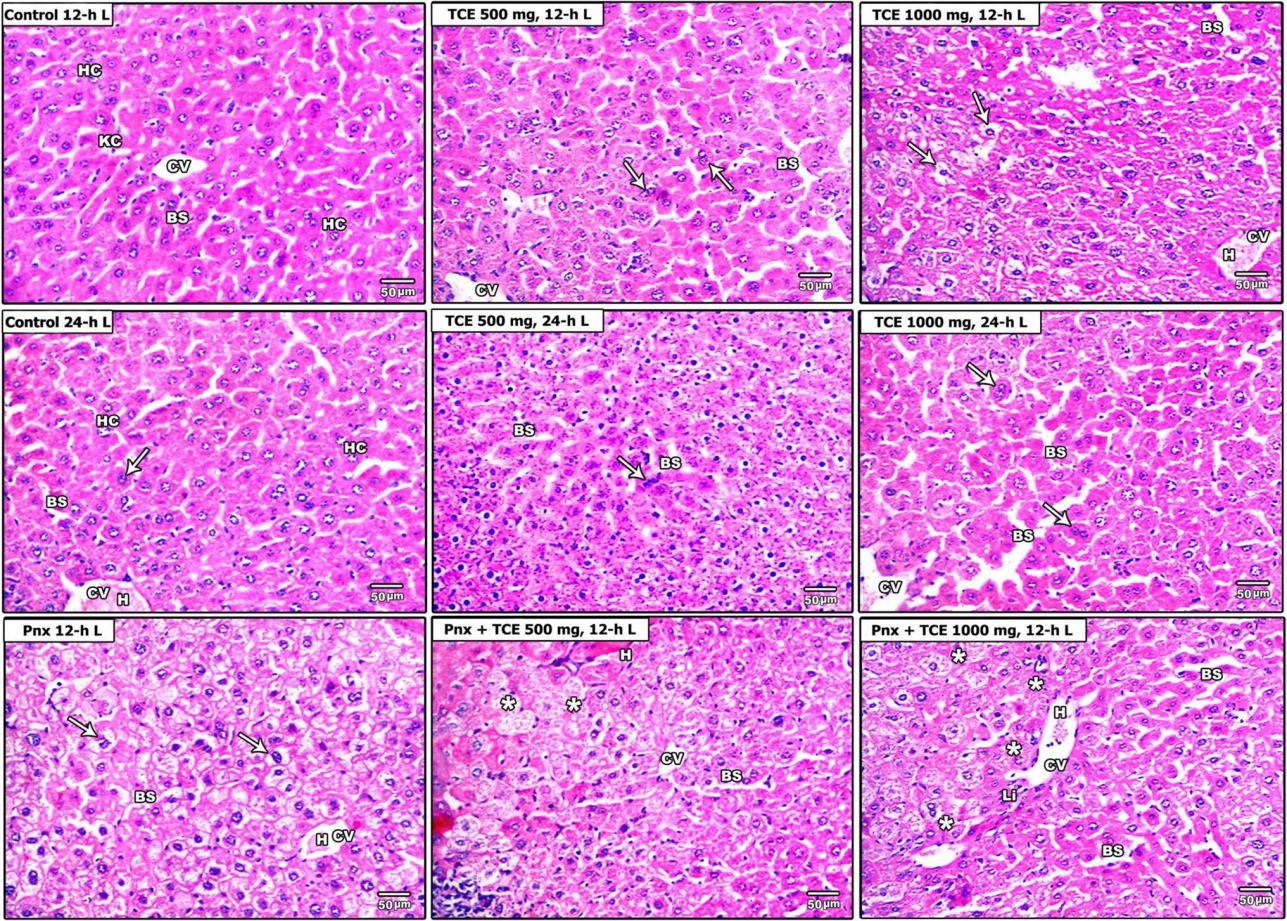

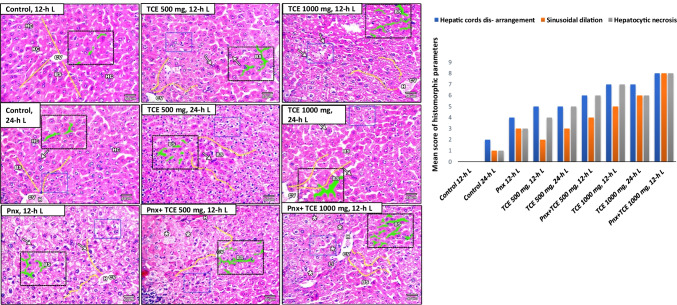
Histopathological changes in liver of mice in the control and different treatment groups **A**. Hepatocytes (HC), central vein (CV), blood sinusoids (BS), Kupffer cells (KC), hemorrhage (H), pyknosis (Pk). Semi-quantitative scoring of hepatic injury **B** represented by hepatic cords arrangement (yellow lines), sinusoidal dilation (green color inside black squire), and hepatocytic necrosis (blue squire). Liver injury parameters were scored as none (0): no involvement of evaluated field; mild (1): involvement of 0–25% of evaluated field; moderate (2): involvement of 25–50% of evaluated field; and severe (3): involvement of 50–100% of evaluated fields in different experimental groups

The livers of 12-h L/12-h D-exposed animals showed normal hepatocytes arranged in cords around the central vein (grade 0 injury) (Fig. 3A, B). On the other hand, the livers of 24-h L-exposed mice showed moderate alteration in the structural organization of the hepatic lobules, hemorrhage, and mild dilation of blood sinusoids. Additionally, mild degree of cellular atypia within hepatocytes was observed (Fig. 3A, B).

The livers of mice subjected to 12-h L/12-h D and treated with TCE 500 mg/kg and the group that exposed to constant light showed moderate degree of cellular atypia within hepatic cells. Sections from the livers of TCE 500 mg, 24-h L mice showed liver damage exhibited by loss of liver architecture, dilation of blood sinusoids, and cellular atypia of eosinophilic foci of affected hepatocytes (grade 5 injury) (Fig. 3A, B).

Pnx mice subjected to 12-h L exhibited marked alteration in liver architecture with diffused hepatic granular vacuolation as well as hemorrhage (grade 4 injury) (Fig. 3A, B). In Pnx, TCE 500 mg, and 12-h L mice, the liver sections displayed clear preneoplastic foci associated with tigrolysis of hepatocytic cytoplasm. This was accompanied by dilation of blood sinusoids with hemorrhage (grade 6 injury). The Pnx, TCE 1000 mg, and 12-h L treated group showed complete deterioration of liver architecture, with massive dilation of both blood sinusoids and central vein with hemorrhage, pyknosis, and ballooning degeneration of hepatocytes as well as leukocytic infiltration. These changes are associated with clear preneoplastic foci that compress the hepatic parenchyma (grade 8 injury).

### Serum expression levels of AFP and VEGF

**Fig. 4:**
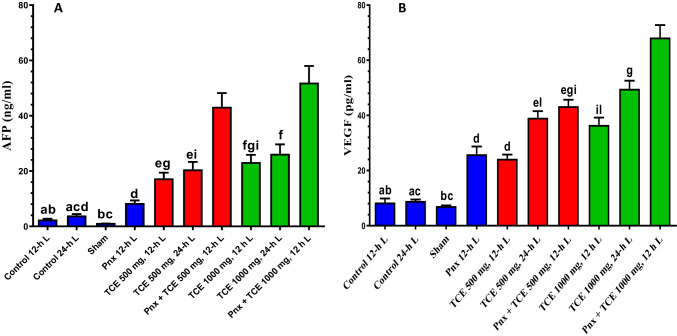
Serum levels of Alpha feto protein (AFP) **A** and vascular endothelial growth factors (VEGF) **B** in mice in the control and different treatment groups. Values are expressed as mean ± standard deviation of the mean. Same letters represent non-significant difference between groups. Differences were considered statistically significant at *P* < 0.05

There were no changes in AFP serum levels of mice kept in constant light compared with the control (12-h L/12-h D cycle). On the other hand, pinealectomized rats showed a significant (*P* < 0.05) increase in serum AFP compared with control animals. Similarly, all animals treated with both doses of TCE and liver in both light regimens as well as Pnx mice that were treated with TCE and kept in 12-h L displayed a significant (*P* < 0.001) increase in serum AFP levels compared with the control groups. The latter group showed higher values of AFP than other TCE-treated groups. The angiogenic factor VEGF was evaluated in serum. The level of VEGF was insignificantly changed in animals exposed to 24-h light compared with the control group (12-h L/12-h D cycle). Pnx mice kept in the normal light–dark cycle showed a significant (*P* < 0.05) increase in levels of VEGF in serum. Similarly, treatment with either dose of TCE caused a significant (*P* < 0.001) increase in VEGF in serum of all animals subjected to 24-h L than the control. Pinealectomy and treatment with TCE showed a significant (*P* < 0.05) increase in the serum level of VEGF compared with other TCE-only treated and the control groups (Fig. 4).

### Immunohistochemical evaluation of CD44 expression

**Fig. 5:**
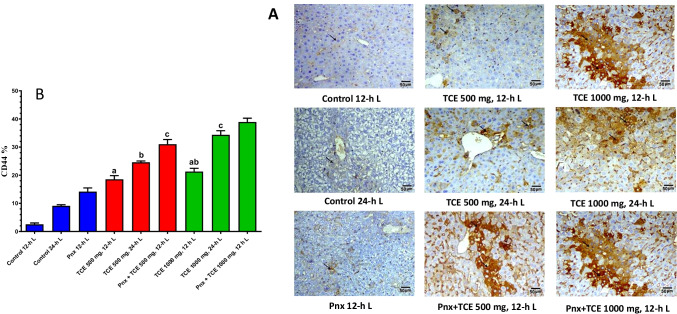
Liver sections of control mice and different treated groups showing **A** immuno-expression of CD44 in the liver. **B** is a quantification of the stained cells. Values are expressed as mean ± standard deviation of 3 microscopic fields/tissue samples. Same letters represent non-significant difference between groups. Differences were considered statistically significant at *P* < 0.05

CD44 is a cancer cell metastasis cell surface protein marker. Therefore, we immunohistochemically determined CD44 expression in mice liver and compared expression levels across the study groups (Fig. 5). A significant increase in the expression of CD44 was observed for all experimental groups compared with control group (12-h L/12-h D cycle). Pinealectomy further elevated the number of CD44-labeled cells in the liver of TCE-treated animals compared with the control group (12-h L/12-h D cycle).

### TGFβ-1 expression profiles

**Fig. 6:**
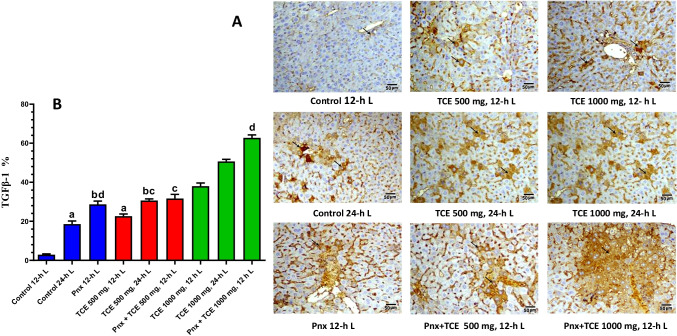
Liver sections of control mice and different treated groups showing **A** immuno-expression of TGFβ-1 in the liver. **B** is a quantification of the stained cells. Values are expressed as mean ± standard deviation of 3 microscopic fields/tissue samples. Same letters represent non-significant difference between groups. Differences were considered statistically significant at *P* < 0.05

Figure 6 shows transforming growth factor beta-1 (TGFβ-1) expression in liver cells. TGFβ-1 expression significantly (*P* < 0.05) increased in all pinealectomized mice as well as TCE-treated groups at both light regimens. Combination of pinealectomy and treatment with TCE showed higher values of TGFβ-1 compared with other groups.

### Liver antioxidant expression levels

**Fig. 7:**
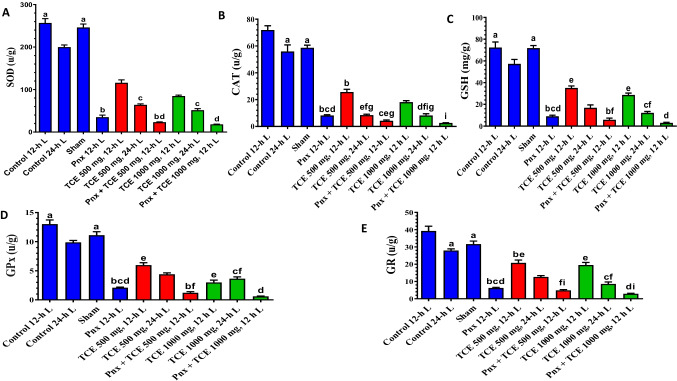
The antioxidants (superoxide dismutase (SOD) activity **A**, catalase (CAT) activity **B**, glutathione (GSH) content **C**, glutathione peroxidase (GPx) **D** activity, and glutathione reductase (GR) activity **E** in the liver of mice in the control and different treatment groups. Values are expressed as mean ± standard deviation of the mean. Same letters represent non-significant difference between groups. Differences were considered statistically significant at *P* < 0.05

We observed a significant decrease in antioxidant enzyme activities (SOD, CAT, GPx, and GR), and GSH concentration in liver of mice kept at 24-h L compared with control mice 12-h L/12-h D (Fig. 7). The Pnx mice maintained 12-h L/12-h D cycle showed a significant (*P* < 0.05) decrease in liver antioxidant levels. There was a significant (*P* < 0.001) decrease in antioxidants in all animals treated with TCE compared with the control mice (Fig. 7). The Pnx mice treated with TCE and maintained in 12-h L/12-h D showed remarkable decrease (*P* < 0.05) in liver antioxidants compared with animals treated only with both doses of TCE and subjected to same light regimen.

### Oxidative stress marker expression profiles

**Fig. 8:**
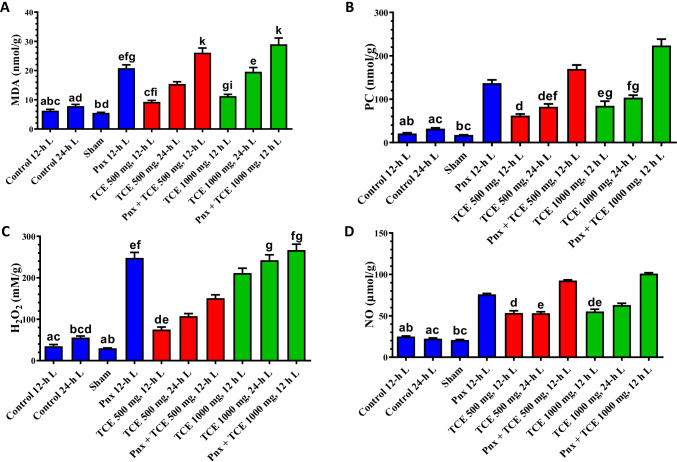
The oxidative stress markers (malondialdehyde (MDA) **A**, protein carbonyl (PC) **B**, hydrogen peroxide (H_2_O_2_) **C**, and nitric oxide (NO) concentrations **D** in the liver of mice in the control and different treatment groups. Values are expressed as mean ± standard deviation of the mean. Same letters represent non-significant difference between groups. Differences were considered statistically significant at *P* < 0.05

Mice exposed to 24-h L exhibited an insignificant change in the oxidative stress markers (H_2_O_2_, MDA, PC, and NO) in liver compared with the control animals (12-h L/12D cycle) (Fig. 8). The Pnx mice maintained 12-h L/12-h D showed a significant (*P* < 0.05) increase in investigated oxidative stress markers. Treatment of animals with either dose of TCE produced a significant (*P* < 0.05) increase in H_2_O_2_, PC, and NO in 24-h L compared with the control mice (12-h L/12-h D). The MDA level showed an insignificant change in the same animals. The Pnx mice treated with TCE and maintained in 12-h L/12-h D showed a remarkable increase (*P* < 0.05) in oxidative markers in the liver compared with animals treated with TCE only with either dose and subjected to the same light regimen (Fig. 8).

### Proinflammatory mediator expression changes

**Fig. 9:**
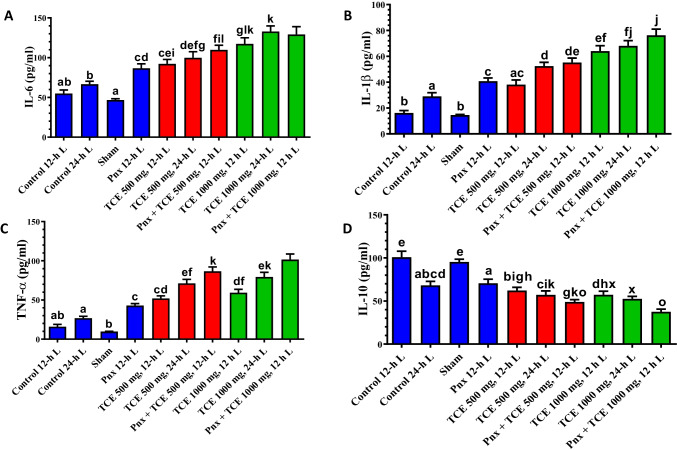
The pro-inflammatory cytokines (IL-6, IL-1β TNF-α) **A**, **B**, **C**, respectively, and anti-inflammatory cytokine (IL-10) **D** in the liver of mice in the control and different treatment groups. Values are expressed as mean ± standard deviation of the mean. Same letters represent non-significant difference between groups. Differences were considered statistically significant at *P* < 0.05

Mice exposed to 24-h L exhibited an insignificant change in the proinflammatory cytokine (IL-6, IL-1β, and TNF-α) expression. However, a significant decrease (*P* < 0.05) in IL-10 was observed in 24-h L compared with control (12-h L/12-D cycle) (Fig. 9). Animals subjected to pinealectomy showed a significant increase in the proinflammatory cytokines and a significant (*P* < 0.05) decrease in IL-10. Treatment of animals with either dose of TCE produced a significant (*P* < 0.05) increase in IL-6, IL-1β, and TNF-α and a significant decrease in IL-10 compared with the control group (Fig. 9). The effect of TCE on the pro-and anti-inflammatory cytokines was more evident in Pnx and TCE-treated mice kept at constant light than in the control animals.

## Discussion

The aim of the present study was to determine the effect of constant light exposure, and pinealectomy on susceptibility to TCE-induced liver toxicity and cancer induction. Our findings suggest that constant light and pinealectomy increase the TCE-induced hepatotoxic effect characterized by expression of cancer markers through upregulation of oxidative stress and inflammation. The findings of this study showed a significant decrease in melatonin levels in the blood of animals subjected to 24-h light or pinealectomy for 30 days compared with the control group (12-h L/12-h D). Exposure to light at night, the time for elevated expression of melatonin, has been reported to inhibit melatonin synthesis and secretion (Touitou et al. 2017).

Exposure to artificial light at night is a source of pollution and a growing public health issue in modern societies. This phenomenon is referred to as light stress because it affects the circadian clock (Touitou and Point 2020) and induces sleep deprivation. The present study revealed that removal of the pineal gland resulted in a significant decrease in serum melatonin levels with increased oxidative stress in the liver and inflammatory cytokines in blood. The increased level of H_2_O_2_ and NO augmented protein oxidation in mice liver. The elevated levels of oxidative markers are attributed to a remarkable decrease in enzymatic (SOD, CAT, GPx, and GR) and non-enzymatic antioxidants (GSH and melatonin) in liver of animals kept in constant light and/or exhibiting pineal impairment (Cichoż-Lach and Michalak 2014). These findings suggest that exposure to constant illumination at night and/or pinealectomy stimulates redox state and the undesirable influences of oxidative radicals.

Oxidative stress is the main causative factor for several liver diseases (Li et al. 2015). The activated redox state influences pathways of inflammatory, metabolic, and proliferative liver diseases (Cichoż-Lach and Michalak 2014). Melatonin is the main antioxidant and free radical scavenger system in the body that clears the buildup of free radicals during the day, mitigating oxidative stress and inflammatory responses and enhancing anti-inflammatory impact under several conditions exposure to ionizing radiation and diabetes (El-Missiry et al. 2020, 2007, 2021). It was recently reported that constant light not only eliminated the circadian rhythms of the expression of the clock genes but also eliminated the circadian rhythms in the genes involved in lipid metabolism in liver and fat cells (Yamamuro et al. 2020). Because circadian rhythm depends on an internal clock under the control of the pineal gland and melatonin level in the blood (Aulinas 2000), it is suggested that constant light and pineal gland impairment are strong chronodisruptors affecting physiological function of the body’s organs, including the liver.

The present study showed that pinealectomy or exposure to constant light resulted in a significant increase in AFP, CD44, TGFβ-1, and VEGF levels indicating early development and progression of hepatic cancer cells. These data were further confirmed by histopathological examination of Pnx and TCE-treated groups, which demonstrated preneoplastic foci and ballooned cells, which are associated with tigrolysis of the hepatic cytoplasm and nuclear atypia. AFP levels have been reported to significantly elevate in liver damage and cancers, and thus, has been used as a tumor marker (Jiang et al. 2018). Emerging evidence has illustrated the role of ROS in stimulating the expression of TGFβ-1, which in turn, in a positive feedback loop, increases ROS generation by diminishing antioxidant enzyme expression (Liu and Desai 2015). The involvement of TGFβ-1 in signaling for cancer occurrence and development is illustrated in several studies (Liu et al. 2019). Meanwhile, melatonin in a dose-dependent manner significantly attenuated the TGFβ-1-dependent stimulation of epithelial-mesenchymal transition in mouse AML12 hepatocyte cells by deactivating ROS signaling (Kim et al. 2019). These data suggest that melatonin is crucial factor for protection against liver cancer development through controlling signaling pathways. While melatonin treatment inhibits tumor growth, metabolism, and proliferation, pinealectomy and/or constant light exposure stimulates tumor growth (Blask et al. 1999). Treatment with melatonin inhibits mammary carcinogenesis in pinealectomized rat kept in the standard light/dark regimen or under constant illumination regimen (Anisimov 2003). Thus, pinealectomy or constant light exposure stimulate adverse health problems and induce cancer development. Therefore, we suggest that light and melatonin are environmental signals that collaborate with each other to control body physiology and regulate tumor initiation and cancer induction. It is also suggested that elevation in oxidative stress and inflammatory cytokine levels due to low melatonin levels might contribute to upregulation of tumor markers in the liver.

It is believed that cancer incidence increased with increased exposure to TCE (Wartenberg et al. 2000). In the present study, TCE exhibited a significant carcinogenic effect in normal (12-h L/12-h D), constant light (24-h L) exposed rats and in pinealectomized rats as well as in pinealectomized rats subjected to TCE compared with normal rats kept in 12-h L/12-h D cycle. The present study demonstrated a severe reduction in melatonin levels with a significant increase in AFP, CD44, TGFβ-1, and VEGF as well as proinflammatory cytokines, including IL-6, IL-1β and TNF-α, with marked decrease in the anti-inflammatory cytokine (IL-10) in these animals. It is reported that TCE increases the differentiation of Th17 cells and increases IL-17 secretion by inducing IL-6 with TGF-β (Shen et al. 2012). The decreased melatonin level after TCE treatment might indicate pineal dysfunction. The combination of pinealectomy and TCE displayed the highest effect on these parameters, which might be attributed to low melatonin levels and increased oxidative stress. TCE and constant light exposure induced remarkable reduction in melatonin levels and antioxidants, with an elevation in oxidants that lead to increased oxidative stress in the liver. Oxidative stress plays a primary role in the starting action of hepatic and extrahepatic damage (Masarone et al. 2018). These results suggest that constant light and pineal ectomy increased mice susceptibility to TCE-induced toxicity and promoted cancer.

TCE and its metabolites can produce liver cancer in mice and put humans at risk of developing liver cancer through several mechanisms, including somatic mutation and modification of cell signaling pathways, but the actual mechanisms involved have not been established (Bull 2000). TCE significantly increases oxidative stress and inflammatory cytokines, with a decline in antioxidant protection and anti-inflammatory (IL-10) levels. These effects are very likely attributable to low melatonin levels. The toxic effect of TCE was more pronounced when combined with constant light and was exuberated by pinealectomy than in the 12-h L/12-h D cycle. This agrees with previous work that TCE can induce an increase in oxidative DNA damage in rat liver (Toraason et al. 1999). It is reported that rats that received Safrole carcinogen at night, when melatonin level is high, exhibited lower DNA damage than animals treated with the same carcinogen during the day (Tan et al. 1994). Circadian variation in TCE toxicity in rodents under different lighting regimens demonstrated increased liver function parameters and necrosis in the hepatocyte in both 12-h L/12-h D and constant darkness (Motohashi et al. 1990). The present results support these findings and showed remarkable increase in the activity of ALT, AST, and bilirubin content with a severe decrease in albumin concentration in blood, indicating liver toxicity. These changes were confirmed using histopathological evaluation.

## Conclusion

The findings from the current study indicate that pineal gland impairment, exposure to continuous light at night, or their combination increase susceptibility to the carcinogenic effects of TCE on the liver. This may be attributable to suppressed antioxidant defenses due to elevated oxidative stress products and inflammatory cytokines in mice.

## Data Availability

All data generated or analyzed during this study are included in this published article.

## References

[CR1] Amer ME, Othamn AI, El-Missiry MA (2021). Melatonin ameliorates diabetes-induced brain injury in rats. Acta Histochem.

[CR2] Amin AH, El-Missiry MA, Othman AI, Ali DA, Gouida MS, Ismail AH (2019). Ameliorative effects of melatonin against solid Ehrlich carcinoma progression in female mice. J Pineal Res.

[CR3] Anggorowati N, Ratna Kurniasari C, Damayanti K, Cahyanti T, Widodo I, Ghozali A, Romi MM, Sari DC, Arfian N (2017) Histochemical and immunohistochemical study of α-SMA, collagen, and PCNA in epithelial ovarian neoplasm. Asian Pac J Cancer Prev: APJCP 18:667–671. 10.22034/APJCP.2017.18.3.667 10.22034/APJCP.2017.18.3.667 PMC5464482 28440973

[CR4] Anisimov VN (2003). The role of pineal gland in breast cancer development. Crit Rev Oncol Hematol.

[CR5] Aulinas A (2000) Physiology of the pineal gland and melatonin. In: Feingold KR et al. (ed.) Endotext. MDText.com, Inc. Copyright © 2000–2021, MDText.com, Inc., South Dartmouth (MA). PMID: 31841296 31841296

[CR6] Blask DE, Sauer LA, Dauchy R, Holowachuk EW, Ruhoff MS (1999). New actions of melatonin on tumor metabolism and growth. Biol Signals Recept.

[CR7] Bull RJ (2000). Mode of action of liver tumor induction by trichloroethylene and its metabolites, trichloroacetate and dichloroacetate. Environ Health Perspect.

[CR8] Burgess HJ, Emens JS (2020). Drugs used in Circadian sleep-wake rhythm disturbances. Sleep Med Clin.

[CR9] Carter BD, Diver WR, Hildebrand JS, Patel AV, Gapstur SM (2014). Circadian disruption and fatal ovarian cancer. Am J Prev Med.

[CR10] Chen Y, Tan F, Wei L, Li X, Lyu Z, Feng X, Wen Y, Guo L, He J, Dai M, Li N (2018). Sleep duration and the risk of cancer: a systematic review and meta-analysis including dose-response relationship. BMC Cancer.

[CR11] Cichoż-Lach H, Michalak A (2014). Oxidative stress as a crucial factor in liver diseases. World J Gastroenterol.

[CR12] Custódio PR, Colombo J, Ventura FV, Castro TB, Zuccari D (2020). Melatonin treatment combined with TGF-β silencing inhibits epithelial-mesenchymal transition in CF41 canine mammary cancer cell line. Anticancer Agents Med Chem.

[CR13] De Miranda BR, Greenamyre JT (2020). Trichloroethylene, a ubiquitous environmental contaminant in the risk for Parkinson's disease. Environ Sci Process Impacts.

[CR14] El-Missiry MA, Fayed TA, El-Sawy MR, El-Sayed AA (2007). Ameliorative effect of melatonin against gamma-irradiation-induced oxidative stress and tissue injury. Ecotoxicol Environ Saf.

[CR15] El-Missiry MA, El-Missiry ZMA, Othman AI (2020). Melatonin is a potential adjuvant to improve clinical outcomes in individuals with obesity and diabetes with coexistence of Covid-19. Eur J Pharmacol.

[CR16] El-Missiry MA, Shabana S, Ghazala SJ, Othman AI, Amer ME (2021). Melatonin exerts a neuroprotective effect against gamma-radiation-induced brain injury in the rat through the modulation of neurotransmitters, inflammatory cytokines, oxidative stress, and apoptosis. Environ Sci Pollut Res Int.

[CR17] Elkin ER, Harris SM, Loch-Caruso R (2018). Trichloroethylene metabolite S-(1,2-dichlorovinyl)-l-cysteine induces lipid peroxidation-associated apoptosis via the intrinsic and extrinsic apoptosis pathways in a first-trimester placental cell line. Toxicol Appl Pharmacol.

[CR18] Elkin ER, Bridges D, Loch-Caruso R (2019). The trichloroethylene metabolite S-(1,2-dichlorovinyl)-L-cysteine induces progressive mitochondrial dysfunction in HTR-8/SVneo trophoblasts. Toxicology.

[CR19] Garcia-Saenz A, de Miguel AS, Espinosa A, Costas L, Aragonés N, Tonne C, Moreno V, Pérez-Gómez B, Valentin A, Pollán M (2020). Association between outdoor light-at-night exposure and colorectal cancer in Spain. Epidemiology.

[CR20] Hansen J, Sallmén M, Seldén AI, Anttila A, Pukkala E, Andersson K, Bryngelsson I-L, Raaschou-Nielsen O, Olsen JH, McLaughlin JK (2013). Risk of cancer among workers exposed to trichloroethylene: analysis of three Nordic cohort studies. J Natl Cancer Inst.

[CR21] Hassan I, Kumar AM, Park HR, Lash LH, Loch-Caruso R (2016). Reactive oxygen stimulation of interleukin-6 release in the human trophoblast cell line HTR-8/SVneo by the trichlorethylene metabolite S-(1,2-Dichloro)-l-Cysteine. Biol Reprod.

[CR22] Horzmann KA, Portales AM, Batcho KG, Freeman JL (2020). Developmental toxicity of trichloroethylene in zebrafish (Danio rerio). Environ Sci Process Impacts.

[CR23] Huang P, Li X, Liu W, Liu J (2015) Advances in non-carcinogenic toxicity of trichloroethylene. Zhonghua Yu Fang Yi Xue Za Zhi 49:844–848. PMID: 26733146 26733146

[CR24] Huang Y, Xia Y, Tao Y, Jin H, Ji C, Aniagu S, Chen T, Jiang Y (2021). Protective effects of resveratrol against the cardiac developmental toxicity of trichloroethylene in zebrafish embryos. Toxicology.

[CR25] Hunter CM, Figueiro MG (2017) Measuring light at night and melatonin levels in shift workers: a review of the literature. Biol Res Nurs 19:365–374. 10.1177/1099800417714069 10.1177/1099800417714069 PMC5862149 28627309

[CR26] Jiang N, Zeng KN, Dou KF, Lv Y, Zhou J, Li HB, Tang JX, Li JJ, Wang GY, Yi SH, Yi HM, Li H, Chen GH, Yang Y (2018). Preoperative alfa-fetoprotein and fibrinogen predict hepatocellular carcinoma recurrence after liver transplantation regardless of the Milan criteria: model development with external validation. Cell Physiol Biochem.

[CR27] Khafaga AF, El-Sayed YS (2018). Spirulina ameliorates methotrexate hepatotoxicity via antioxidant, immune stimulation, and proinflammatory cytokines and apoptotic proteins modulation. Life Sci.

[CR28] Khafaga AF, Noreldin AE, Taha AE (2019). The adaptogenic anti-ageing potential of resveratrol against heat stress-mediated liver injury in aged rats: Role of HSP70 and NF-kB signalling. J Therm Biol.

[CR29] Kim JY, Park JH, Kim K, Leem J, Park KK (2019). Melatonin inhibits transforming growth factor-β1-induced epithelial-mesenchymal transition in AML12 hepatocytes. Biology (basel).

[CR30] Li S, Tan HY, Wang N, Zhang ZJ, Lao L, Wong CW, Feng Y (2015). The role of oxidative stress and antioxidants in liver diseases. Int Mol Sci.

[CR31] Liu H, Zhu Y, Zhu H, Cai R, Wang KF, Song J, Wang RX, Zhou RX (2019). Role of transforming growth factor β1 in the inhibition of gastric cancer cell proliferation by melatonin in vitro and in vivo. Oncol Rep.

[CR32] Liu RM, Desai LP (2015). Reciprocal regulation of TGF-beta and reactive oxygen species: a perverse cycle for fibrosis. Redox Biol.

[CR33] Maganhin CC, Simoes RS, Fuchs LF, Oliveira-Filho RM, Simoes Mde J, Evencio Neto J, Baracat EC, Soares JM (2009). Rat pinealectomy: a modified direct visual approach. Acta Cir Bras.

[CR34] Manouchehri E, Taghipour A, Ghavami V, Ebadi A, Homaei F, Latifnejad Roudsari R (2021). Night-shift work duration and breast cancer risk: an updated systematic review and meta-analysis. BMC Womens Health.

[CR35] Masarone M, Rosato V, Dallio M, Gravina AG, Aglitti A, Loguercio C, Federico A, Persico M (2018). Role of oxidative stress in pathophysiology of nonalcoholic fatty liver disease. Oxid Med Cell Longev.

[CR36] Motohashi Y, Kawakami T, Miyazaki Y, Takano T, Ekataksin W (1990). Circadian variations in trichloroethylene toxicity under a 12:12 hr light-dark cycle and their alterations under constant darkness in rats. Toxicol Appl Pharmacol.

[CR37] Noreldin AE, Gewaily MS, Saadeldin IM, Abomughaid MM, Khafaga AF, Elewa YH (2021) Osteoblast-activating peptide exhibits a specific distribution pattern in mouse ovary and may regulate ovarian steroids and local calcium levels. Am J Trans Res 13:5796–5814. PMID: 34306327 PMC8290782 34306327

[CR38] Phillips RV, Rieswijk L, Hubbard AE, Vermeulen R, Zhang J, Hu W, Li L, Bassig BA, Wong JYY, Reiss B, Huang Y, Wen C, Purdue M, Tang X, Zhang L, Smith MT, Rothman N, Lan Q (2019). Human exposure to trichloroethylene is associated with increased variability of blood DNA methylation that is enriched in genes and pathways related to autoimmune disease and cancer. Epigenetics.

[CR39] Reiter RJ, Rosales-Corral SA, Tan DX, Acuna-Castroviejo D, Qin L, Yang SF, Xu K (2017). Melatonin, a full service anti-cancer agent: inhibition of initiation, progression and metastasis. Int J Mol Sci.

[CR40] Schernhammer ES, Laden F, Speizer FE, Willett WC, Hunter DJ, Kawachi I, Fuchs CS, Colditz GA (2003). Night-shift work and risk of colorectal cancer in the nurses' health study. J Natl Cancer Inst.

[CR41] Shen T, Wang J, Xu H, Xu SH, Jiang T, Zhu QX (2012) Effect of trichloroethylene intake via drinking water on Th17 cells in BALB/c mice. Zhonghua Yu Fang Yi Xue Za Zhi 46:152–157. PMID: 22490199 22490199

[CR42] Stenvers DJ, van Dorp R, Foppen E, Mendoza J, Opperhuizen AL, Fliers E, Bisschop PH, Meijer JH, Kalsbeek A, Deboer T (2016). Dim light at night disturbs the daily sleep-wake cycle in the rat. Sci Rep.

[CR43] Sulli G, Lam MTY, Panda S (2019). Interplay between Circadian clock and cancer: new frontiers for cancer treatment. Trends Cancer.

[CR44] Talib WH (2018). Melatonin and cancer hallmarks. Molecules.

[CR45] Tan D, Reiter RJ, Chen LD, Poeggeler B, Manchester LC, Barlow-Walden LR (1994). Both physiological and pharmacological levels of melatonin reduce DNA adduct formation induced by the carcinogen safrole. Carcinogenesis.

[CR46] Toraason M, Clark J, Dankovic D, Mathias P, Skaggs S, Walker C, Werren D (1999). Oxidative stress and DNA damage in Fischer rats following acute exposure to trichloroethylene or perchloroethylene. Toxicology.

[CR47] Touitou Y, Reinberg A, Touitou D (2017). Association between light at night, melatonin secretion, sleep deprivation, and the internal clock: health impacts and mechanisms of circadian disruption. Life Sci.

[CR48] Touitou Y, Point S (2020). Effects and mechanisms of action of light-emitting diodes on the human retina and internal clock. Environ Res.

[CR49] Ukawa S (2018). Association between average daily television viewing time and the incidence of ovarian cancer: findings from the Japan Collaborative Cohort Study. Cancer Causes Control.

[CR50] van Zuylen ML, Meewisse AJG, Ten Hoope W, Eshuis WJ, Hollmann MW, Preckel B, Siegelaar SE, Stenvers DJ, Hermanides J (2021). Effects of surgery and general anaesthesia on sleep-wake timing: CLOCKS observational study. Anaesthesia.

[CR51] Walker WH, Bumgarner JR, Walton JC, Liu JA, Melendez-Fernandez OH, Nelson RJ, DeVries AC (2020). Light pollution and cancer. Int J Mol Sci.

[CR52] Wall AC, Gius JP, Buglewicz DJ, Banks AB, Kato TA (2019). Oxidative stress and endoreduplication induced by blue light exposure to CHO cells. Mutat Res Genet Toxicol Environ Mutagen.

[CR53] Wartenberg D, Reyner D, Scott CS (2000). Trichloroethylene and cancer: epidemiologic evidence. Environ Health Perspect.

[CR54] Wu C, Schaum J (2000). Exposure assessment of trichloroethylene. Environ Health Perspect.

[CR55] Yamamuro D, Takahashi M, Nagashima S, Wakabayashi T, Yamazaki H, Takei A, Takei S, Sakai K, Ebihara K, Iwasaki Y, Yada T, Ishibashi S (2020). Peripheral circadian rhythms in the liver and white adipose tissue of mice are attenuated by constant light and restored by time-restricted feeding. PLoS ONE.

[CR56] Zisapel N (2018). New perspectives on the role of melatonin in human sleep, circadian rhythms and their regulation. Br J Pharmacol.

